# Biocompatible Materials for Orbital Wall Reconstruction—An Overview

**DOI:** 10.3390/ma15062183

**Published:** 2022-03-16

**Authors:** Victor A. Vasile, Sinziana Istrate, Raluca C. Iancu, Roxana M. Piticescu, Laura M. Cursaru, Leopold Schmetterer, Gerhard Garhöfer, Alina Popa Cherecheanu

**Affiliations:** 1 Department of Ophthalmology, Faculty of Medicine, Carol Davila University of Medicine and Pharmacy, District 5, 020021 Bucharest, Romania; victor-andrei.vasile@drd.umfcd.ro (V.A.V.); alina.cherecheanu@umfcd.ro (A.P.C.); 2 Department of Ophthalmology, University Emergency Hospital, 020021 Bucharest, Romania; 3 Nanostructured Materials Laboratory, National R&D Institute for Nonferrous and Rare Metals, 077145 Pantelimon, Romania; roxana.piticescu@imnr.ro (R.M.P.); mpopescu@imnr.ro (L.M.C.); 4 Singapore National Eye Centre, Singapore Eye Research Institute, Singapore 168751, Singapore; leopold.schmetterer@seri.com.sg; 5 Ophthalmology and Visual Sciences Academic Clinical Program, Duke-NUS Medical School, National University of Singapore, Singapore 169857, Singapore; 6 SERI-NTU Advanced Ocular Engineering (STANCE), Singapore 639798, Singapore; 7 School of Chemical and Biological Engineering, Nanyang Technological University, Singapore 637459, Singapore; 8 Department of Clinical Pharmacology, Medical University Vienna, 1090 Vienna, Austria; gerhard.garhoefer@meduniwien.ac.at; 9 Center for Medical Physics and Biomedical Engineering, Medical University Vienna, 1090 Vienna, Austria; 10 Institute of Molecular and Clinical Ophthalmology, 4056 Basel, Switzerland

**Keywords:** orbital reconstruction, biocompatible materials, 3D printing, orbital implant

## Abstract

The reconstruction of an orbit after complex craniofacial fractures can be extremely demanding. For satisfactory functional and aesthetic results, it is necessary to restore the orbital walls and the craniofacial skeleton using various types of materials. The reconstruction materials can be divided into autografts (bone or cartilage tissue) or allografts (metals, ceramics, or plastic materials, and combinations of these materials). Over time, different types of materials have been used, considering characteristics such as their stability, biocompatibility, cost, safety, and intraoperative flexibility. Although the ideal material for orbital reconstruction could not be unanimously identified, much progress has been achieved in recent years. In this article, we summarise the advantages and disadvantages of each category of reconstruction materials. We also provide an update on improvements in material properties through various modern processing techniques. Good results in reconstructive surgery of the orbit require both material and technological innovations.

## 1. Introduction

### 1.1. Causes of Orbital Fracture

Craniofacial fractures frequently involve the orbit [1]. Orbits are conical structures with irregular walls that surround and protect the eyeballs and separate the upper segment of the facial skeleton from the middle one [2]. Although the conical shape helps maintain the position of the eyeballs when accelerating, it does not provide protection in the case of deceleration injuries [3]. The most common causes of orbital fractures among the adult population are domestic violence and motorcycle accidents, while among children, the most common causes are sports accidents and falls [4].

### 1.2. Types of Orbital Fractures

There are several types of orbital fractures: medial or lateral wall fractures; orbito-zygomatic fractures; LeFort I, II, and III fractures; and naso-orbital ethmoid fractures (Markowitz fractures) [5]. The most common are orbital floor fractures, medial wall fractures, and zygomatic fractures [6]. The surgical approach in the case of orbital floor fractures can be either conjunctival, cutaneous, or trans-antral exposure [7,8]. Trans-maxillary and trans-nasal endoscopic approaches have also been described [9]. Hypoglobus and enophthalmos are the most common major complications that can appear after orbital trauma, and these are due to the enlargement of the orbital volume [10,11]. Primary and secondary reconstruction of the orbit are currently aided by computer-assisted pre-operative planning and intraoperative navigation [12,13].

### 1.3. Management of Orbital Fracture

Managing orbital fractures is difficult because of the impact they can have on vision. Their treatment must be preceded by a thorough clinical evaluation and concomitant management of associated eye lesions [14,15]. Clinical and radiological evaluation of the traumatized area helps establish the need for surgery [16,17]. Such fractures can lead to deformities and affect vision, but such side-effects can also occur after surgery, so proper surgery and the right choice of material for reconstruction are essential [4,18]. There are concerns about wear-induced debris, as material-related complications need to be addressed when choosing the best implant [19,20]. Complications such as diplopia and enophthalmos can represent indications for surgery [5,21]. In 29% of cases, orbital fractures are associated with eye damage [22]. A complete clinical evaluation from an ophthalmological point of view must include: determination of visual acuity and intraocular pressure, biomicroscopic examination of the anterior and posterior pole, assessment of the visual field, ocular and pupil motility, and an external examination [4].

## 2. Biomaterials for Reconstruction

Over time, several types of biomaterials have been used for osteosynthesis following traumatic fractures, osteotomies, or for filling bone defects [23]. Titanium alloy microplates were preferred due to their biocompatibility, stability, and easy adjustment to the affected area, but the eventual need for reintervention for plate and screw extraction raised the issue of using bioresorbable alternatives [24]. The ideal biomaterial for reconstruction in craniofacial fractures must meet certain criteria of biocompatibility, stability, safety, intraoperative adjustment, and low cost [25]. There are several types of biomaterials used for osteosynthesis, including metals (stainless steel—very rarely used today, cobalt alloys, titanium) and resorbable osteosynthesis materials (polylactic acid—PLA, polyglycolic acid—PGA, polyhydroxybutyrate, and poly-methyl-carbonate) [26]. The materials used for bone replacement are bone tissue (autologous bone tissue, allograft, xenogenic bone tissue, and demineralized bone tissue) and alloplastic materials (metals, ceramic materials, plastics, and combinations of materials with different mechanical and osteoconductive properties) [24]. The most common implant materials used in orbital reconstruction are bone and cartilage autografts, alloplasts, such as titanium mesh, porous polyethylene, resorbable sheeting, and patient-specific implants [27]. Each of these materials has specific indications, advantages, and disadvantages [4] (Table 1).

### 2.1. Bone

Autologous bone tissue became the gold standard in reconstructing bone defects in the 18th century. Tissue can be harvested from the iliac crest, calvaria, maxilla, mandible, tibia, scapula, and sternum [28].

Allogeneic bone tissue is harvested from a human donor and subjected to inactivation, denaturation, and sterilization procedures [29]. These operations result in an exclusively inorganic bone matrix that is subsequently implanted. Other types of bone tissue replacement are xenografts and demineralized bone tissue from the human donor [24].

Split calvaria grafts are commonly used to reconstruct orbital fractures [30]. Results of the studies conducted in the past 10 years showed that these grafts are safe and lead to a reduction in diplopia and enophthalmos [31]. However, calvaria grafts are not as good at providing a precise recovery of the orbital volume [4]. Kontio et al. conducted a study on 24 patients regarding orbital reconstruction using a free iliac bone graft. They obtained a low rate of enophthalmos and hypophthalmos and an 80% rate of bone resorption [4]. In 1999, Kosaka et al. described the mandibular bone grafting for the treatment of orbital blowout fractures with the following advantages: (1) ease of harvesting, (2) ease of thinning, (3) grafts of appropriate size and shape available, (4) postoperative immobilization not necessary, and (5) absence of visible scars [32]. In 2004, Kosaka et al. conducted a study on 75 patients regarding the efficacy of a bone grafting from the mandibular outer cortex to reconstruct the orbital walls and concluded that the mandibular outer cortical plate is the first choice grafting material for the treatment of orbital blowout fractures with several advantages, including (1) the absence of functional disability, (2) no secondary deformity, (3) the absence of postoperative difficulties with respect to breathing and walking, and (4) major complications are rare [32].

Bone tissue has good strength, can be fixed to adjacent bones, and is radio-opaque, but has a variable degree of resorption and cannot be easily modeled, which can become problematic in orbital fractures. The main disadvantages of using autologous bone tissue in craniofacial reconstructions are, on the one hand, the morbidity at the level of the graft and, on the other hand, the increased duration and complexity of the surgery [33]. Bone autografts are used in pediatric fractures (less than seven years of age) because of their good strength, best biocompatibility, lack of sharp edges, and their radio-opaque property [34]. However, the surgeon may find difficulties in adjusting them to shape, donor site morbidity is significant, the operation is time-consuming and expensive, and bone resorption may lead to complications [4].

### 2.2. Cartilage

Autologous cartilage tissue can also be used to reconstruct the orbital floor because it is easily accessible, malleable, and no signs of resorption have been described in association with these implants [35]. Grafts are harvested more frequently from the ear canal and the nasal septum [36]. The advantage of harvesting cartilaginous tissue from the nasal septum is that the harvesting time is short, and the morbidity and cosmetic damage are minimal [28]. In 2009, Bayat et al. performed a randomized clinical trial on 22 patients with blowout fractures and found that the patients treated with a nasal septal cartilage graft had significantly better correction of enophthalmos than those treated with conchal cartilage (*p* = 0.02) after 10 days (*p* = 0.02), 1 month (*p* = 0.004), and 3–6 months (*p* = 0.001) [37].

The use of cartilage autografts has the same disadvantages as bone. Moreover, cartilage is not radio-opaque and does not provide as good a support as bone autografts. This type of material is indicated in small orbital fractures and is most frequently harvested from the nasal septum, leading to minimal donor site morbidity but also leaving the patient without the option of having nasal surgery [4].

### 2.3. Alloplastic Materials

Alloplastic materials available for reconstruction in craniofacial fractures include titanium, ceramics (bioactive ceramic glass, calcium phosphate, hydroxyapatite, tricalcium β phosphate, aluminum oxide, calcium sulfate), and plastics (acrylates, porous polyethylene) [24]. Implants made of alloplastic materials such as titanium mesh, hydroxyapatite, and porous polyethylene (Medpor) ensure good tensile strength but have the possibility of infections, bleeding, or migration of the implant [38].

#### 2.3.1. Metals

Titanium is the most biocompatible and corrosion-resistant metal. It is chemically similar to calcium, making it physiologically inert [28]. The coefficient of elasticity of titanium corresponds to that of bone tissue better than that of any other metal [39]. Titanium is found in the lithosphere, but obtaining pure titanium (with less than 1% additives) is difficult and expensive, consisting of its extraction from iron ore by complex processes. Osteosynthesis materials are generally titanium alloys that may contain low amounts of aluminum, vanadium, or niobium. The toxicity of titanium is very low, but it can be increased by alloy additives (aluminum can accumulate in cases of kidney failure, and it is neurotoxic) [40]. Currently, titanium is available in several forms: plates (miniplates, microplates), nets, or screws. In present times, modular plates are preferred at the expense of rectangular plates that were used in the past [24].

Titanium plates and screws have a satisfactory strength and elasticity for rigid fixation of the fractured bone during the healing period and are biologically inert [41]. In addition, titanium attaches to adjacent bones and frequently remains asymptomatic [42,43]. Titanium-containing implants can become infected, migrate, be expelled, cause pain, foreign body reaction, or even kidney failure by accumulating corrosion products [44]. In such cases, surgery is performed to remove the implant. On the other hand, in asymptomatic cases, the eventual removal of the implant after healing is controversial [24].

Regarding the use of titanium to fill large defects in orbitozygomatic or orbitofrontal reconstructions, the problem of subsequent implant removal does not raise an issue because large defects require permanent stability, and the extraction of plates and nets would be much more laborious. Titanium mesh has been approved since 1984 and been used in craniomaxillofacial surgery, especially for major defects [28]. Titanium nets of various thicknesses are available, each type serving different purposes. Thin nets with a thickness of 0.1 mm (M-TAM, Stryker Leibinger) can be individually shaped, cut with scissors, and fixed with screws. They can be used for primary fractures with bone loss. Titanium micro nets have been used successfully in anterior wall fractures of the frontal sinus and have allowed re-pneumatization. Thicker meshes (0.3–0.6 mm) can be used in case of contour irregularities [24].

Titanium mesh implants individually bent were used for primary reconstruction of the orbital floor after orbital trauma with satisfying results regarding volume and shape [45]. They were inserted using a retroseptal transconjunctival approach and fixed with 1.0 or 1.3 titanium micro-screws [46]. The position of such implants can be visualized in cone-beam computed tomography (CBCT), which reduces radiation compared to the use of standard CT examination [12].

Titanium implants used in craniofacial reconstructions are traditionally manufactured in standard shapes, which means that during surgery, they are manually adapted to the anatomical shape of the patient’s bone defect. This manual adaptation to the anatomical shape of the patient’s bone during surgery is time-consuming and a source of error in the strict adaptation to the patient’s bone defect, especially for surgeons who do not have much experience [47]. Additionally, manual adaptation involves multiple manipulations of the implant, which leads to increased internal mechanical stress of the implant. This results in many clinical complications, including implant rupture, corrosion, weakening of the screws, and bone resorption [48,49,50].

Shaping and bending titanium mesh plates can be challenging and can lead to errors [51]. As a result, another technique called direct metal laser sintering was imagined to prevent this issue. It helps create a customized titanium mesh [52]. To prevent enophthalmos, a mirroring reconstruction technique was used in orbital floor reconstruction. This led to obtaining a virtual design mesh that was patient-specific [53].

#### 2.3.2. Resorbable Osteosynthesis Materials

Disadvantages of using titanium implants for osteosynthesis include the need for surgical reintervention in case of complications, skull growth in children that can lead to translocation of the implant, sensitivity to low temperatures, and imaging interference, which have led to the need for the development of alternative solutions: bioresorbable polymers [42,54]. Polymers are large molecules formed by the repetition of subunits. They can be classified into resorbable and non-resorbable, and porous and non-porous, respectively [28,55].

Medpor (ultra-high-density porous polyethylene) is a non-resorbable, easy-to-shape polymer that has been frequently used in small orbital floor defects [56]. This material has a smooth surface and very good biocompatibility because the pores allow the formation of connective tissue and blood vessels [57]. The results obtained with Medpor were similar to those obtained with autologous bone tissue, but infection rates were lower [28].

Porous polyethylene has also proven its utility in reconstructing defects with good edges to support the implant [58]. It has good strength and can be adjusted well to the defect. The disadvantages of using porous polyethylene are the costs and the fact that it does not allow egress of fluid from the orbit [4].

Although their properties were first studied in the 1960s, polymer-based bioresorbable osteosynthetic materials began to be widely used in the 1990s. This category includes polyglycolic acid (PGA), poly-L lactic acid. (PLLA), poly-D lactic acid (PDLA), unsynthesized hydroxyapatite, and copolymers PGA, PLLA, and PDLA [42]. Although biodegradable polymers are considered biocompatible, their resorption produces a foreign body-like reaction. Cases of fistulas, osteolysis, and soft tissue edema following the resorption of these osteosynthesis materials have also been described [24]. Moreover, in the case of orbital fractures, the results obtained with resorbable polymers were not favorable—the complication rate was high [28].

In addition to eliminating the risk of reintervention, resorbable polymers also have other advantages: lack of metal corrosion, lack of radiological interference, and lower incidence of osteopenia [59]. However, the difficult adjustment and bending of resorbable polymer plates, as well as their controversial strength, have turned researchers’ attention towards magnesium [60]. Magnesium alloys have been used in pediatric orthopedics and vascular surgery in the past for their degrading properties, but they form excess hydrogen and are not particularly resistant, which is why their use has been abandoned [61]. Recently, several studies aimed to solve these problems. Such publications showed that adding aluminum to magnesium alloys improves their strength. However, further research is needed to neutralize the disadvantages of magnesium (hydrogen production, high corrosion, and low biocompatibility) [42].

#### 2.3.3. Material Combinations

There are many possible combinations using the materials mentioned above. For example, titanium-reinforced porous polyethylene sheets can be used for complex orbital defects [62]. The role of titanium is to ease fixation into the bone, as it makes the implant’s manipulation more precise. Moreover, titanium makes the implant radiovisible [4].

#### 2.3.4. Patient-Specific Implants

Patient-specific implants are digitally designed implants based on the contralateral orbit, which can be used to reconstruct complex and extensive orbital fractures [63]. They have the advantages of being biocompatible, radio-opaque, and more stable than manually bent titanium [64]. They can be placed at a specific location with intraoperative CT guidance [65]. On the other hand, creating the implant is time-consuming and expensive, and it requires an intact contralateral orbit [4].

In recent years, more and more studies have reported the use of 3D-printed patient-specific implants [66]. Li et al. reported 18 cases of maxillomandibular reconstruction using 3D printed implants and 8 cases of maxillofacial reconstruction using 3D-printed patient-specific titanium implants in orthognathic surgery. The study showed that the patient-specific implants had a significant improvement in morphology, particularly in large and complex-shaped defects [48]. In 2014, Stoor et al. presented a study of 12 orbital reconstructions (including both orbital and maxillary reconstructions) using 3D-printed patient-specific Ti6AI4V ELI implants of thickness and size adapted to the bone defect (between 0.5 and 0.8 mm) [67]. Most implants were placed through a subciliary incision and fixed with 2 mm screws [68]. The study showed a shortening of the surgery time (1.17 h using patient-specific implants, correspondingly 1.57 h using an intraoperative bending technique) but also that two patient-specific implants (16%) had a false shape due to incorrect CAD because data of thin bone did not transfer correctly to CAD and resulted in an error. Stoor et al. suggested that this “thin bone phenomena” can possibly be solved in the future by using the morphometry of the airspace in the opposite maxillary sinus instead of the bony structure. Rotaru et al. presented a series of 10 reconstructions of the calvaria using 3D printed titanium implants analyzing both the degree of symmetry and the complications [48,69]. The study found that the difference between the volume of the reconstructed right calvaria and the left calvaria was not statistically significant, while the aesthetic appearance was much improved [69].

Many studies have been published in recent years regarding comparing 3D-printed titanium implants and those manufactured as standard and manually adapted during an operation [70]. For example, Wilde et al. compared the two types of implants in terms of biomechanical properties and concluded that 3D printed ones offer superior stability and rigidity [48]. However, the main advantage of customized 3D printed implants is the shortening of the surgery time and implicitly in shortening the anesthesia and reducing its risks, as well as the accuracy of adapting the implant to the bone defect with the restoration of orbital volume, which determines a better functional result from the point of view of ocular motility as well as binocular vision [71]. These conclusions are also supported by Zimmerer et al. and Fan et al. [72,73,74].

Thus, in the light of current results, the use of 3D-printed patient-specific implants seems to be the best option for orbital reconstruction, especially in those involving large bone defects.

#### 2.3.5. Resorbable Sheeting

From this category of implant materials, resorbable sheeting has been successfully used to reconstruct fractures with small gaps and stable lateral and medial borders. Resorbable sheeting is sheets made of poly-L/D-lactide, polyglactin, and polydioxanone. They have also proven useful in pediatric orbital fractures. Some authors recommend its use for defects <2.5 cm due to the loss of long-term structural support [75]. Resorbable sheeting is pliable and can be adjusted to the defect. The advantages of resorbable sheeting are wide availability, good maneuverability in the wound, the possibility of intraoperative contouring, and smooth surface and edges. Their disadvantages include their cost, the fact that they are not radiopaque (postoperative implant cannot be viewed), degradation of the material can cause contour loss, sterile inflammation, serous orbit drainage is less efficient than in titanium meshes, and long-term stability and support [4,76].

Polycaprolactone (PCL) is a biodegradable polyester that has some advantages: it is hydrophobic and slow-degrading (up to two years) and has also been used in combination with HAP to promote cell adhesion to the surface of the material [77]. The PCL implant has been used for large defects (over 20 mm horizontal width defect) with as good results as in small defects, and new bone formation visualized on CT scans 1.5 years after implantation [78]. Complications related to fibrovascular integration into the porous implant may increase the risk of restriction and diplopia due to cicatrization between the porous implant and the orbital soft tissue [79].

## 3. Recent Discoveries

Recent discoveries regarding the reconstruction of the craniomaxillofacial skeleton include the use of recombinant bone morphogenetic protein (recombinant BMP) in combination with various biomaterials such as demineralized bone, bioresorbable synthetic polymers to support their integration, surface changes of implants to promote their osseointegration, bifocal distraction osteogenesis, and tissue engineering [24].

Because excessive wear and premature degradation can adversely affect the biocompatibility of materials used to reconstruct various types of fractures, preventing healing and having long-term negative effects, the emphasis has recently been on improving their properties [80]. Thus, these materials can be loaded with biological factors, such as bone morphogenetic protein-2 (BMP-2), transforming growth factor-β (TGF-β), fibroblasts, platelet and vascular endothelial growth factors (FGF, PDGF, VEGF), and others, to stimulate cell attachment and biocompatibility, or to release necessary molecules and ions during the biodegradation of supporting materials (Table 2). An example is magnesium which, as it degrades, releases ions that have stimulating effects on the generation of new bone tissue [81].

**Figure 1:**
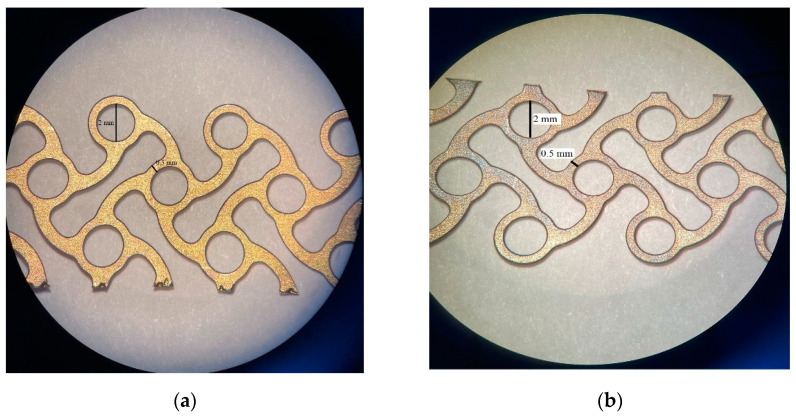
Titanium mesh surfaces—coated with hydroxyapatite for orbital wall reconstruction: (**a**) Microscopic image of the top face; (**b**) Microscopic image of the bottom face.

Changing the surface of these implants is one of the most common strategies to improve biocompatibility. The addition of surface roughness and porosity stimulates cell attachment and osseointegration [81] (Figure 1).

**Figure 2:**
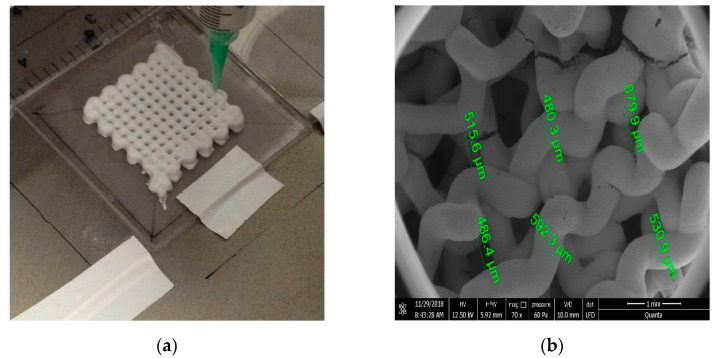
Hydroxyapatite scaffolds for bone tissue engineering made by 3D printing: (**a**) Macroscopic view; (**b**) Detailed view of the interconnecting channel structure with diameter of about 500 µm.

**Figure 3:**
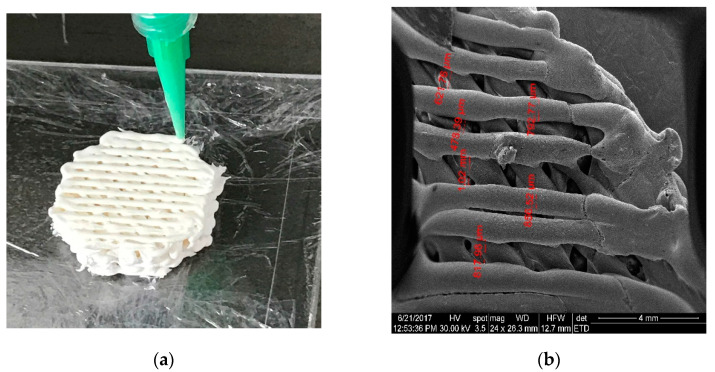
Hydroxyapatite scaffolds for bone tissue engineering made by 3D printing: (**a**) Macroscopic view; (**b**) Detailed view of the interconnecting channel structure with diameter of about 800 µm.

Additive manufacturing has been used lately for surface modification of different biomaterials: metals, ceramics, and polymers (Figure 2 and Figure 3). There are two types of surface modifications that can be used on reconstruction materials to enhance their biocompatibility and their performances in general: physical modifications and chemical modifications. Physical modifications include grit-blasting, machining, and etching, and these lead to changes in the morphology or topography of the surface. Chemical techniques include plasma and chemical vapor and electro–chemical or atomic layer deposition and can result in single or multiple layer coatings using different compounds, oxidizing, nitriding or carbiding a surface, ion infusion, and functionalization of a surface [83].

## 4. Surface Modifications of Metallic Biomaterials, Ceramics, and Polymers Using Additive Manufacturing

Metal implants are not an ideal environment for cell adhesion because they are smooth and have low wettability [84]. To enhance tissue integration for metallic biomaterials, additive manufacturing was used to create roughness and obtain a porous structure in the outermost layer while trying to preserve a dense structure on the inside. Compared to flat titanium, implants obtained after additive manufacturing have led to better mineralization in vitro [83].

However, these changes in surface topography could also affect the interactions between pathogenic microorganisms and surfaces. In terms of metallic biomaterials, 3D printing seems to be a promising method of modifying their architectures to increase biocompatibility [85]. However, this method increases the contact surface between the implant and tissues with increased ion release from the implant. Hence, there is the need to add a ceramic or polymer film to the surface of the 3D implant to limit the release of ions [81].

The main modification used for ceramics is polymeric coating. It helps enhance the implant’s integrity in vivo and maintain steady release profiles, but the surface modification of ceramics occurs mostly in the field of resorbable drug-delivery devices. One of the biggest challenges is the inability to sinter an AM ceramic after it has been loaded with drugs because of the low degradation temperature of the additional material. As an alternative, additives such as trace elements (Mg, Sr, Si, Zn) found in normal tissue are used as methods to increase osteogenesis and angiogenesis [83].

In all polymer constructions, often without major changes to the design file of the device, the starting material composition can be modified to induce nano- and microtopography, variation in chemical composition, and even crystallography [83]. Hydroxyapatite, a calcium phosphate, was used in some studies with polymers [76,86]. This led to the formation of a polymer-based material with nano-sized and fully crystallized ceramic granules at its surface [87,88]. This technique made the implant rough and provided it with a chemical structure and crystallography similar to bone [83].

## 5. Prospects and Future Prospects

There are a few anatomical regions in the human body that are as controversial as the type of biomaterial to use in orbit reconstruction: resorbable versus non-resorbable, autogenous/autologous/xenogenic versus alloplastic, titan mesh standard versus an adapted one, non-porous versus porous materials, and coated versus non-coated. Because of the assorted challenges in orbital reconstruction, currently, there is no ideal biomaterial suitable for all scenarios. New-generation biomaterials are expected to cart a significant added value not only in terms of biocompatibility, bioactivity, and bone-regenerating ability but also in terms of the ability to act as matrices for in situ drug delivery [89]. Despite technological advances in bone development, new materials and methods of bone healing continue to be investigated [90]. The limiting factors of bone graft substitutes currently in use show that further improvements are needed [85].

## 6. Conclusions

The reconstruction of an orbit after complex fractures can be extremely challenging because of the impact they can have on vision. The most common implant materials used in orbital reconstruction are bone and cartilage autografts, alloplasts such as titanium mesh, porous polyethylene, resorbable sheeting, and patient-specific implants. Each of these materials has specific indications, advantages, and disadvantages. Good results in reconstructive surgery of the orbital walls require both material and technological innovations.

## Figures and Tables

**Table 1:** Implant material indications [4].

	Implant Material	Indication
Autograft	Bone	Pediatric fractures (<7 years of age)
	Cartilage	Small orbital fractures
Alloplast	Resorbable sheeting	Pediatric fractures
	Porous polyethylene	Defects with solid edges
	Titanium mesh	Large defect of the orbital floor Small gaps with stable lateral and medial borders
	Patient-specific implant	Complex and extensive orbital defects

**Table 2:** Biological factors and their effect on biocompatibility [82].

Biological Factors	Effect
Transforming growth factor β 1 (TGF-β1)	× regulates bone remodeling
Bone morphogenetic protein-2 (BMP-2)	× strong osteoinductive effect
Vascular endothelial growth factors (VEGFs)	× stimulates the formation of new blood vessels × used for vascular materials as well as for bone regeneration
Fibroblast growth factors (bFGF or FGF-2)	× pro-angiogenic role × increases cell proliferation

## Data Availability

No new data were created or analyzed in this study. Data sharing is not applicable to this article.
